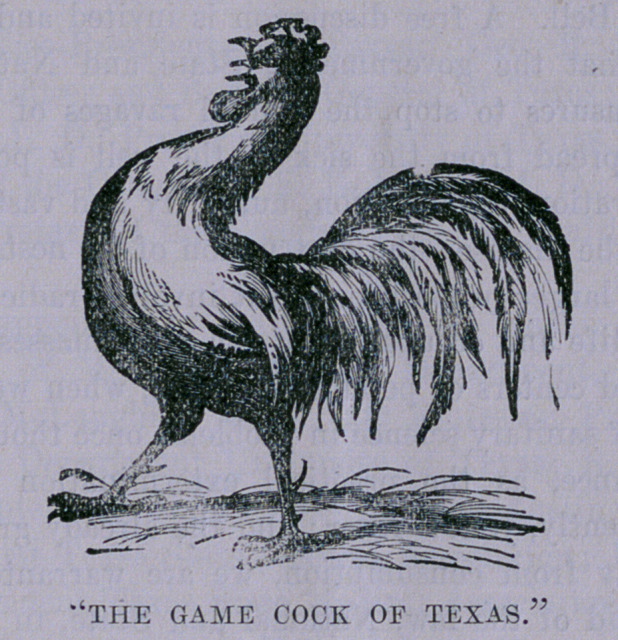# The American International Congress on Tuberculosis — Fourth Biennial Session, New York City, November 14, 15, 16, 1906

**Published:** 1906-08

**Authors:** 


### EDITORIAL DEPARTMENT.

"THE GAME COCK OF TEXAS."

### The American International Congress on Tuberculosis —Fourth Biennial Session, New York City, November 14, 15, 16, 1906.

The Congress will be held under the patronage of the Federal government. In our last issue we published the letter of Secretary of State Root to the American ambassadors and consuls in foreign countries, instructing them to extend invitations to the respective governments to which they are accredited, to send delegates to the Congress. Many responses have been received, and a large and enthusiastic attendance of distinguished sanitarians and other scientists is assured. The Congress will be opened by the Medico-Legal Society of America, Hon. Clark Bell, LL. D., President. Addresses will be delivered by distinguished men selected for the occasion, after which the President will deliver his inaugural address. Programs will be ready in due time and a bureau of information will be opened at 39 Broadway for the benefit of members, delegates, and visitors. A special reduced rate of travel and for hotels will be secured and duly announced.

The main object of the Congress will be to arouse a public sentiment for preventive measures to restrict the spread of the disease from the sick to the well, and for the removal of the causes of the disease, so far as it may be done, by reform in building and the enforcement of sanitary measures. One of the most important questions for discussion and action will be, "What legislation is needed to carry into effect the objects of this Congress; and how can it best be secured"? This question will be presented in a paper* by Hon. Clark Bell. A free discussion is invited and expected.

It is time that the governments, State and National, should take active measures to stop the fearful ravages of consumption. To limit the spread from the sick to the well is possible, but it requires co-operation, organization, authority and vast means. The extirpation of the disease—the destruction of its nests— its breeding places, is a larger proposition, as it involves radical reforms in the manner of life and occupation of the great masses of humanity in our congested centers of population. Yet, when we reflect upon the conquests of sanitary science in problems once thought Utopian, such, for instance, as the practical extermination of smallpox, plague, and recently, yellow fever; and the already great reduction in the mortality from consumption, we are warranted in hoping that with the aid of the law, National and State, in the course of time the disease will have been practically suppressed,—if not entirely "stamped out." And when we reflect that of the (about) 80,000,000 of people in America, probably 8,000,000 or 10 per cent are *already doomed;* and that for every death from yellow fever (past record) there are one hundred and fifty from consumption; and further, that in the same length of time, say four years, there are more lives lost by consumption, a preventable disease, than during the Civil war, counting all causes on both sides, it would seem to require no argument to convince the general government of its imperative duty in the premises. The daily average of deaths in America, from consumption, is four hundred and eleven—every day—all the time!

*This paper appears in last issue of the *Medico-Legal Journal* and in the *Texas Medical News* for July (ult.).

## Figures and Tables

**Figure f1:**